# Status of initial treatment for rapidly progressive glomerulonephritis in Japan: analysis of a personal clinical records database

**DOI:** 10.1007/s10157-025-02657-0

**Published:** 2025-03-27

**Authors:** Joichi Usui, Tomonori Kimura, Kunihiro Yamagata, Kentaro Nakajima, Shuzo Kaneko, Ken-ei Sada, Naotake Tsuboi, Hirokazu Okada, Keiju Hiromura, Yoshitaka Isaka, Ichiei Narita

**Affiliations:** 1 Department of Nephrology, Institute of Medicine, University of Tsukuba, Tsukuba, Ibaraki 305-8575 Japan; 2 Department of Nephrology, Osaka University Graduate School of Medicine, Suita, Japan; 3 Department of Nephrology, Itabashi Central General Hospital, Tokyo, Japan; 4 Department of Clinical Epidemiology, Kochi Medical School, Nankoku, Japan; 5 Department of Nephrology, School of Medicine, Fujita Health University, Toyoake, Japan; 6 Department of Nephrology, Faculty of Medicine, Saitama Medical University, Saitama, Japan; 7 Department of Nephrology and Rheumatology, Gunma University Graduate School of Medicine, Maebashi, Japan; 8 Division of Clinical Nephrology and Rheumatology, Kidney Research Center, Niigata University Graduate School of Medical and Dental Sciences, Niigata, Japan

**Keywords:** RPGN, ANCA, Anti-GBM antibody, Personal clinical records database

## Abstract

**Background:**

As a joint project with the Ministry of Health, Labour and Welfare (MHLW), the Research Group on Intractable Renal Diseases is examining the feasibility of utilizing its personal clinical records database. We examine the validity of the initial-treatment data from the personal clinical records of patients with new-onset rapidly progressive glomerulonephritis (RPGN).

**Methods:**

Personal clinical records for patients with either RPGN or anti-glomerular basement membrane (GBM) antibody nephritis were used. The data from 454 newly enrolled RPGN patients were compiled for analysis in 2 cohort studies (CS1 for all case analysis and CS2 for selective analysis of new-onset cases).

**Results:**

In CS1, the serotypes of the 362 registered RPGN cases included 200 myeloperoxidase (MPO)-ANCA-positive, 98 anti-GBM-positive, and 9 proteinase-3 (PR3)-ANCA-positive cases, etc. CS2 included 96 of the MPO-ANCA-positive RPGN and 55 of the anti-GBM antibody-positive RPGN cases. For the initial treatment of MPO-ANCA-positive RPGN, the rates of glucocorticoid (GC) and GC pulse treatment were similar between the personal clinical records database and the nationwide questionnaire survey, but the rates of intravenous cyclophosphamide (CY) or rituximab were statistically significant lower in the personal clinical records database. For the initial treatment of anti-GBM antibody-positive RPGN, the rate of plasma exchange was similar between the two databases, but the rates of GC and per os CY tended to be lower in the personal clinical records database, although not statistically significant.

**Conclusion:**

Clear differences in initial treatment for new-onset RPGN patients were found between a personal clinical records database and another reported database.

## Introduction

In Japan, a nationwide questionnaire survey to determine the status of medical practice for rapidly progressive glomerulonephritis (RPGN), a representative intractable renal disease, has been conducted since 1996 [1]. Using this nationwide survey, data on diagnosis, treatment, and prognosis have been gathered and used as a reference for the preparation of clinical practice guidance or guidelines (CPGs) [2, 3]. Although this nationwide survey cannot deny the existence of the selection bias because the method of implementation is a questionnaire, over time, public awareness campaigns conducted in Japan in regard to RPGN and the establishment of norms for its diagnosis and treatment have contributed to an improvement in the vital prognosis of RPGN in general as well as that for cases of antineutrophil cytoplasmic antibody (ANCA)-associated RPGN, which is a major subset of RPGN. In Western countries, evidence-based practice guidelines recommend the use of cyclophosphamide (CY), an immunosuppressant, or rituximab (RTX), a biological agent, in combination with glucocorticoid (GCs) as the standard initial treatment for ANCA-associated RPGN [4, 5]. However, the majority of patients with new-onset ANCA-associated RPGN in Japan start treatment with GC monotherapy, indicating a gap between the evidence obtained from clinical trials in Western countries and the current status of treatment in Japan [2]. In the early days of RPGN treatment in Japan, GC was often used as the sole treatment. In addition to this, it is undeniable that the treatment guideline for ANCA-associated RPGN has been to control vasculitis while avoiding infection and death, which may have influenced the frequency of GC monotherapy [6]. Considering these actual practices in Japan, unlike European guidelines, Japanese clinical practice guidelines (both RPGN and AAV) include the option of GC monotherapy [3, 7]. Furthermore, the use of immunosuppressive drugs and biologics with Japanese patients has increased in recent years, while the proportion of patients treated with GC alone has decreased, closing the gap with Europe. Therefore, it is necessary to understand the relationship between the standard treatment in Western countries and the real-world treatment regimens in Japan, as well as changes in these regimens over time and their relation to disease prognosis, to consider future treatment recommendations.

In Japan, following the establishment of the Act on Medical Care for Patients with Intractable Diseases (hereinafter referred to as the Act on Intractable Diseases) in 2014, a medical expense aid program was initiated in January, 2015. To receive medical expense aid, patients need to consult designated physicians to prepare a medical certificate, i.e., a personal clinical record that qualifies a patient to apply for grants for specific medical expenses. These personal clinical records, which are managed by the Ministry of Health, Labour and Welfare (MHLW), have been partly compiled into a database and are expected to be utilized in epidemiologic studies [8]. In this study, we analyzed a subset of data derived from this database i.e., the clinical records of patients with RPGN or anti-glomerular basement membrane (GBM) antibody nephritis to verify its validity for use in epidemiologic studies of intractable disease. At the same time, we sought to clarify the status of the initial treatment for RPGN.

## Materials and methods

### The personal clinical records database of intractable disease

This cross-sectional study used data from a nationwide administrative database of public expenditures for refractory diseases, i.e., the National Database of Designated Intractable Diseases of Japan, which includes data on RPGN and anti-GBM antibody nephritis, maintained by MHLW [8]. The records consist of prospectively and annually collected clinicopathological data from Japanese patients. The data were registered only after review by certified nephrologists as Medical Certificates of Designated Intractable Diseases. The database does not include survival data. Details of the data curation have been described previously [9].

The number of patients applying for each serological type of RPGN was calculated using the personal clinical records database by isolating entries for the designated intractable diseases: RPGN (entry no. 220) and anti-GBM antibody nephritis (entry no. 221). (https://www.nanbyou.or.jp/entry/235, https://www.nanbyou.or.jp/entry/4379, in Japanese). Records lacking a description of the patient birth date, disease onset date and serological disease type are not included in the clinical records database, and so they were automatically excluded from our analysis as well. However, the clinical personal records database did include patients who had been followed-up for long duration. Since we were focused on new-onset patients, we excluded all patients for whom disease onset occurred more than one year prior to medical-certificate application. As a result, two cohort studies were established as follows: Cohort study 1 (CS1) for all case analysis, and CS2 for selective analysis of new-onset cases. CS2 was limited to cases in which the year of medical-certificate application and the year of onset were the same. The number of patients with each serological disease type was calculated for the number of patients who applied. Next, for each serological disease type, we identified age, gender, RPGN clinical grading score (score I-IV), [10] renal pathological findings (necrotizing crescentic glomerulonephritis), chronic kidney disease (CKD) stage (G1-5, A1-3), and treatment including medication [GC, intravenous GC pulse, intravenous CY (IVCY), per os CY (POCY), RTX, methotrexate, mycophenolate mofetil, azathioprine, mizoribine, cyclosporine, and other immunosuppressive drugs], plasma exchange, and chronic dialysis. Each table was accompanied by data from the nationwide questionnaire survey (2016–2017) as a comparative control (unpublished data); this questionnaire survey is considered the standard for used in the research of RPGN in Japan [2].

### Statistical analyses

Qualitative data are presented as the number of cases and percentage (%), and quantitative data are presented as the mean and standard deviation (SD). The χ^2^ test was used to compare qualitative data between the two groups, CS2 and nationwide questionnaire survey. The Mann–Whitney U-test was used to compare quantitative data. All presented p-values were two-sided tests, and p-values < 0.05 were accepted as significant. IBM SPSS Statistics ver. 29.0 (IBM, Armonk, NY) was used for the statistical analyses.

### Ethics approval and consent to participate

The study complies with the Declaration of Helsinki. The original study protocol was approved by the ethics review board of the Japanese Society of Nephrology (No. 70, December 10, 2019). The present sub-study was approved by the ethics review board of the University of Tsukuba Hospital (No. R1-352, March 3, 2020). The requirement for informed consent was waived because all data included in this dataset were de-identified. This study was conducted in accordance with MHLW regulations. The study protocol of the nationwide questionnaire survey used for comparison with the present data was approved by the ethics review board of the University of Tsukuba Hospital (No. R4-158, October 27, 2022). The analysis data of this study differs from the statistics and other data prepared and published by the Ministry of Health, Labour and Welfare of Japan.

## Results

**Fig. 1:**
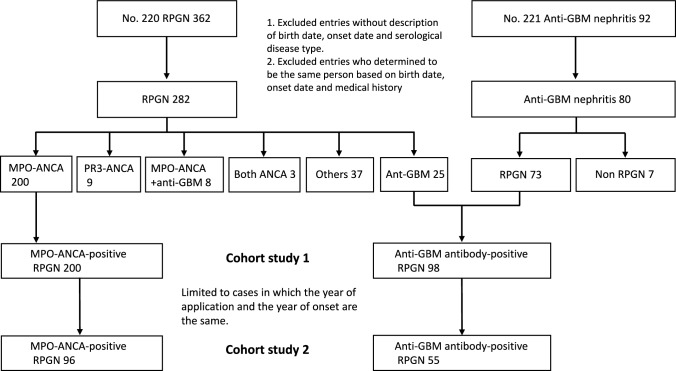
Inclusion and exclusion criteria of this study

In CS1, the serological disease type of the 362 RPGN cases registered in the personal clinical records database for the designated intractable diseases RPGN (No. 220) and anti-GBM antibody nephritis (No. 221) included 200 MPO-ANCA-positive cases, 98 anti-GBM antibody-positive cases, 9 PR3-ANCA-positive cases, 8 cases positive for both MPO-ANCA and anti-GBM antibody, 3 cases positive for both PR3- and MPO-ANCA, and 37 others (Fig. 1).

Next, we separately analyzed the CS2 cohort to focus on initial treatment data for patients with new-onset RPGN. Because there were a sufficient number of entries for two types of RPGN i.e., MPO-ANCA-positive RPGN and anti-GBM antibody-positive RPGN—we included these two types in CS2. Comparing between the data in CS1 and CS2, the similarity between the CS1 data and the questionnaire survey data was greater than the similarity between the CS2 data and the questionnaire survey data (Tables 1, 2). In terms of the clinical characteristics of the two types of RPGN, the data of age, gender and RPGN clinical grading score were similar between those in the CS2 and the nationwide questionnaire survey. Among cases with MPO-ANCA-positive RPGN, there tended to be fewer CKD G stage 5 cases than in the nationwide questionnaire survey, suggesting that some cases of severe renal failure were not registered. On the other hand, CKD G5 cases accounted for the majority of registered cases having anti-GBM antibody-positive RPGN (89.1%), suggesting that such cases with severe renal failure were included in the registry. In MPO-ANCA-positive RPGNs, the frequency of pulmonary involvements with and without pulmonary involvement was comparable and not statistically significantly different between those in the CS2 and the nationwide questionnaire survey.

**Table 1:** Clinicopathological characters and treatments of MPO-ANCA-positive RPGN

	Cohort study 1	Cohort study 2	Nationwide questionnaire survey	**p-value**
Number of cases	200	96	326	
Clinicopathological characters				
Age (average ± SD)	67.3 ± 14.8	70.7 ± 12.5	72.2 ± 12.5 (n = 319)	0.08
Gender (male/female)	80/120	39/57	149/170	0.29
RPGN clinical grading score	57/98/26/13 (n = 194)	24/46/17/8 (n = 95)	65/173/69/11	0.15
Necrotizing glomerulonephritis in histology	133, 97.1% (n = 137)	61, 98.4% (n = 62)	NA	NA
CKD G stage (1/2/3a/3b/4/5)	1/6/8/40/77/64 (n = 196)	0/0/1/13/40/41 (n = 95)	7/19/18/48/91/136	0.08
CKD A stage (1/2/3)	13/42/139 (n = 194)	2/11/82(n = 95)	NA	NA
Lung involvement	66, 35.9% (n = 184)	40, 44.9% (n = 89)	150, 46.9% (n = 320)	0.75
Treatments				
Glucocorticoid	188, 99.5% (n = 189)	94, 100.0% (n = 94)	318, 97.5% (n = 326)	0.13
Intravenous methylprednisolone pulse	72, 38.1% (n = 189)	54, 57.4% (n = 94)	200, 61.3% (n = 326)	0.50
Intravenous cyclophosphamide	8, 4.2% (n = 189)	3, 3.2% (n = 94)	47, 14.4% (n = 326)	< 0.01
Per os cyclophosphamide	7, 3.7% (n = 189)	7, 7.4% (n = 94)	8, 2.5% (n = 326)	0.02
Rituximab	4, 2.1% (n = 189)	3, 3.2% (n = 94)	31, 9.5% (n = 326)	0.048
Methotrexate	0, 0% (n = 189)	0, 0% (n = 94)	NA	NA
Mycophenolate mofetil	2, 1.1% (n = 189)	1, 1.1% (n = 94)	NA	NA
Azathioprine	10, 5.3% (n = 189)	1, 1.1% (n = 94)	NA	NA
Mizoribine	15, 7.9% (n = 189)	0, 0% (n = 94)	NA	NA
Cyclosporin	5, 2.6% (n = 189)	1, 1.1% (n = 94)	NA	NA
Tacrolimus	2, 1.1% (n = 189)	0, 0% (n = 94)	NA	NA
Plasma exchange	9, 4.6% (n = 195)	5, 5.4% (n = 93)	25, 7.7% (n = 326)	0.45
Renal replacement therapy	24, 12.6% (n = 191)	14, 15.4% (n = 91)	69, 21.2% (n = 326)	0.22
Chronic dialysis	15, 62.5% (n = 24)	9, 64.3% (n = 14)	51, 15.6% (n = 326)	< 0.01

P-values are based on comparisons between cohort study 2 and nationwide questionnaire survey

*RPGN* rapidly progressive glomerulonephritis, *NA* not available, *CKD* chronic kidney disease

**Table 2:** Clinicopathological characters and treatments of anti-GBM antibody-positive RPGN

	Cohort study 1	Cohort study 2	Nationwide questionnaire survey	**p-value**
Number of cases	98	55	30	
Clinicopathological characters				
Age (average ± SD)	64.6 ± 16.3	66.6 ± 15.5	61.5 ± 15.2 (n = 30)	0.07
Gender (male/female)	38/60	20/35	12/18	0.74
RPGN clinical grading score	3/8/5/8 (n = 24)	1/0/3/4 (n = 8)	8/16/6/0	0.19
Necrotizing glomerulonephritis in histology	49, 98.0% (n = 50)	20, 100.0%(n = 20)	NA	NA
CKD G stage (1/2/3a/3b/4/5)	0/0/2/7/13/75 (n = 97)	0/0/0/3/3/49	0/0/0/0/7/23	0.72
CKD A stage (1/2/3)	5/10/80 (n = 95)	0/4/50 (n = 54)	NA	NA
Treatments				
Glucocorticoid	67, 90.5% (n = 74)	40, 88.9% (n = 45)	29, 96.7% (n = 30)	0.22
Intravenous methylprednisolone pulse	43, 58.1% (n = 74)	38, 84.4% (n = 45)	24, 80.0% (n = 30)	0.62
Intravenous cyclophosphamide	10, 13.5% (n = 74)	5, 11.1% (n = 45)	3, 10.0% (n = 30)	0.88
Per os cyclophosphamide	5, 6.8% (n = 74)	4, 8.9% (n = 45)	6, 20.0% (n = 30)	0.17
Rituximab	4, 5.4% (n = 74)	3, 6.7% (n = 45)	0 (n = 30)	0.15
Methotrexate	0, 0% (n = 74)	0, 0% (n = 45)	NA	NA
Mycophenolate mofetil	0, 0% (n = 74)	0, 0% (n = 45)	NA	NA
Azathioprine	3 4.1% (n = 74)	1, 2.2% (n = 45)	NA	NA
Mizoribine	1, 1.4% (n = 74)	0, 0% (n = 45)	NA	NA
Cyclosporin	1, 1.4% (n = 74)	0, 0% (n = 45)	NA	NA
Tacrolimus	0, 0% (n = 74)	0, 0% (n = 45)	NA	NA
Plasma exchange	75, 79.8% (n = 94)	43, 81.1% (n = 53)	24, 80.0% (n = 30)	0.90
Renal replacement therapy	16, 75.0% (n = 22)	5, 83.3% (n = 6)	20, 66.7% (n = 30)	0.42
Chronic dialysis	12, 75.0% (n = 16)	5, 100.0% (n = 5)	15, 50.0% (n = 30)	< 0.01

P-values are based on comparisons between cohort study 2 and nationwide questionnaire survey

*RPGN* rapidly progressive glomerulonephritis, *NA* not available, *CKD* chronic kidney disease

In terms of the initial treatment data for MPO-ANCA-positive RPGN, the rates of GC (100% vs. 97.5%) and GC pulse (57.4% vs. 61.3%) were similar between CS2 and the questionnaire survey, while the rates of IVCY (3.2% vs. 14.4%) and RTX (3.2% vs. 9.5%) were statistically significant lower in CS2. On the other hand, the frequency of chronic dialysis (64.3% vs. 15.6%) was statistically significant much higher in CS2. In terms of initial treatment for anti-GBM antibody-positive RPGN, plasma exchange (81.1% vs. 80.0%) was performed at similar rates between CS2 and the questionnaire survey, while GC (88.9% vs. 96.7%) and POCY (8.9% vs. 20.0%) tended to be performed at lower rates in CS2. Again, chronic dialysis was (100% vs. 50.0%) was statistically significant performed more frequently in CS2.

## Discussion

Comparison between the personal clinical records database and the results of the nationwide questionnaire survey was conducted on patients with RPGN and those with anti-GBM antibody nephritis to analyze the validity of the utilization of the clinical records database in epidemiologic studies. From these sources, the real-world status of the clinical picture and initial treatment regimens for MPO-ANCA-positive RPGN and anti-GBM antibody-positive RPGN were identified. The clinicopathological profile and initial treatment of patients with new-onset RPGN identified using the personal clinical records database were different for some items compared to continuously reported survey data, as discussed below.

First, the validity of the utilization of the medical certificate-records database was verified. In the intractable disease program, patients with severe conditions are candidates for medical expense aid in general; applications for patients with mild conditions are limited. Therefore, it is presumed that the personal clinical records are biased in terms of disease severity, and it is unknown to what degree these personal clinical records provided for medical expense aid are representative of patients with intractable disease as a whole. With regard to the registration of RPGN, we assume that all cases are defined as severe at the time of initial onset or relapse, and that the possibility of unregistered mild cases is limited (https://www.nanbyou.or.jp/entry/235, https://www.nanbyou.or.jp/entry/4379, in Japanese). In addition, while some diseases may not require patients with low treatment costs to apply for the designated intractable disease aid, it is common for patients presenting with RPGN to apply because treatment costs for this disease are typically burdensome. Therefore, the personal clinical records for RPGN are less likely to suffer form this omission of mild cases. We compared the personal clinical records and results of a nationwide questionnaire survey for almost the same period, and found that the clinical findings, including renal function and treatment contents, were similar at different points between the two groups. It was clear that few severe cases, including cases of severe renal failure, were registered. The reason for this may be that there is a separate medical subsidy for end-stage kidney disease (ESKD) cases in Japan, and the necessity to apply for the designation of intractable disease has been eliminated by this subsidy. Therefore, ESKD cases might not be registered in the personal clinical records database. In this study, utilization of the personal clinical record database in epidemiologic studies was considered in regard to only two categories of RPGN, MPO-ANCA-positive RPGN and ani-GBM antibody-positive RPGN. A similar comparative analysis of the databases of other intractable diseases is required, and the results of those analyses are expected to expand the utilization of personal clinical record databases in epidemiologic studies.

Second, some differences in the initial treatment for the two types of RPGN, MPO-ANCA-positive RPGN and anti-GBM antibody-positive RPGN, were identified. GC administration and GC pulse in MPO-ANCA-positive RPGN patients, and PLEX in patients with anti-GBM antibody-positive RPGN are almost equally common between the registry and survey, because these are typically done from the very beginning of treatment [3, 4, 11]. The medical-certificate application form for a designated intractable disease describes the treatment up to that point of application. Therefore, it is practical to describe the implementation of a treatment regimen in the midst of its implementation. The timing of application for designated intractable diseases is not consistent among medical institutions and physicians; treatment regimens implemented after the application, possibly including the drug or drugs for which financial aid is required, are not reflected in the database, resulting in underestimation of the number of patients with MPO-ANCA-positive RPGN who received additional IVCY or RTX. Likewise, the use of additional GC administration and POCY for anti-GBM antibody-positive RPGN patients was likely underestimated. The timing of the application also reflects the fact that chronic dialysis is overestimated with respect to prognosis, as the course of treatment includes patients who withdraw from dialysis after the application for designated intractable disease. To overcome these issues, it is necessary to set the time of medical-certificate application for designated of intractable diseases as the time of completion of initial treatment (3–6 months after the start of treatment), and to analyze continuous data utilizing the data at the time of renewal.

In the initial treatment for ANCA-associated glomerulonephritis in Japan, our analysis indicated that almost all patients receiving GC administration, with a smaller number of patients also receiving concomitant immunosuppressants such as CY or the biological agent RTX. In the CPGs based on the results of clinical intervention trials, mainly those conducted by the European Vasculitis Study Group, the use of immunosuppressants or biological agents in combination with GC has been proposed as the standard treatment. However, this recommendation clearly does not correspond with the real-world selection of initial treatment in Japan [2, 4, 5]. There are several sources of this discrepancy: the age group of patients with ANCA-associated glomerulonephritis in Japan is inclined to the elderly (the mean age is in the 70 s year old) and monotherapy with GC is considered an acceptable option, which tends to be chosen because of concerns for the occurrence of infectious disease or serious drug-related complications using the other agents, the cost and convenience. Although vital prognosis in patients with ANCA-associated glomerulonephritis has shown improvement over time in Japan, further improvement in renal prognosis is desired [2]. It is possible that more aggressive introduction of concomitant immunosuppressants or biological agent use would lead to further improvement in renal prognosis in patients with ANCA-associated glomerulonephritis in Japan. This issue should be considered to prevent more patients from entering ESKD status.

The actual status of implementation of PLEX in patients with ANCA-associated glomerulonephritis presents another issue. Worldwide CPGs, including that in Japan, recommend concomitant use of PLEX in patients with severe renal failure [12, 13]. However, in actual clinical practice, the use of PLEX is limited to patients with severe renal failure for whom PLEX is begun immediately. Since treatment with dialysis can be used as an alternative therapy in patients with severe renal failure, the rate of patients in whom PLEX is concomitantly provided may be low because of concerns regarding expensive medical costs or adverse events, such as allergic reaction to blood preparation [2]. Although PLEX has been recommended in CPGs in Japan, concomitant use of PLEX for patients with ANCA-associated glomerulonephritis was only recently included (in 2018) in health insurance, postdating the survey period of this study. Therefore, the number of patients who receive PLEX as combination therapy is expected to have increased in more recent years. It is therefore necessary to confirm the rate of patients who have received PLEX as combination therapy in recent years.

In the personal clinical records of other intractable diseases, No. 43 microscopic polyangiitis (MPA) and No. 44 granulomatous polyangiitis (GPA), contain a larger number of patients with RPGN than the No. 220 RPGN questionnaire used in the present study. Although the point of the possibility that renal limited vasculitis (RLV) is more common in No. 220 RPGN than No. 43 MPA and No. 44 GPA may be raised, we cannot distinguish between RLV and systemic vasculitis including MPA and GPA based on the current No. 220 clinical records. Regarding the extraction of RLV, we will consider modifying the personal clinical record form for use as research data. However, it is possible to confirm the presence or absence of pulmonary involvements, and 44.9% of the patients enrolled in this study had pulmonary involvements. The frequency of pulmonary involvements in the nationwide RPGN questionnaire survey, which is the subject of this comparison, was 46.9%, a similar frequency and not significantly different between them. Therefore, it seems unlikely that systemic vasculitis is more infrequent and RLV is much more frequent. Since the utilization of the personal clinical records of other intractable vasculitis will require a joint survey with Japan Research Committee of the MHLW for Intractable Vasculitis (JPVAS, https://www.vas-mhlw.org/en/index.html), the utilization of individual data for other diseases is a future issue.

A certain level of validity for use of the personal clinical records database in epidemiologic studies is warranted, if the typical application timing for each intractable disease is considered, along with continuity data analysis using data updates. Actually, the possibility of following up data from the same patients using a continuing record is currently being explored. Such evidence can help policy makers evaluate the actual clinical picture and treatment regimens of patients with designated intractable diseases. Additionally, if this project of utilizing the survey form is continued, it can be used to identify changes in treatment options over time. We will consider it as a future research topic.
